# Editorial: Aggression in sports and education

**DOI:** 10.3389/fpsyg.2026.1952016

**Published:** 2026-08-24

**Authors:** Alexandra Predoiu, Georgeta Pânişoară, Andrzej Piotrowski, Radu Predoiu

**Affiliations:** 1 Faculty of Physical Education and Sport, Sports and Motor Performance Department, National University of Physical Education and Sports, Bucharest, Romania; 2 Faculty of Psychology and Educational Sciences, University of Bucharest, Bucharest, Romania; 3 Institute of Psychology, University of Gdańsk, Gdańsk, Poland; 4 Faculty of Physical Education and Sport, Teachers' Training Department, National University of Physical Education and Sports, Bucharest, Romania

**Keywords:** aggression, emotion regulation, resilience, safeguarding, school ecology, sport psychology

Sport and education are widely recognized as powerful settings for promoting physical, psychological, and social development. They foster learning, cooperation, resilience, and wellbeing; however, they also remain contexts in which diverse forms of aggression continue to emerge. Bullying, interpersonal violence, abuse toward officials, cyber aggression, and hostile behaviors challenge the educational and developmental potential of both domains. Contemporary perspectives increasingly recognize aggression as the product of interactions between personal dispositions and environmental influences. The General Aggression Model proposes that aggressive behavior results from the dynamic interplay of personal and situational inputs, internal cognitive and emotional states, and behavioral appraisal processes (Anderson and Bushman, 2002). This perspective suggests that aggression cannot be fully understood without considering the contexts in which behavior develops.

The studies included in this Research Topic support this ecological interpretation. Although conducted across different populations and settings, they converge on a common conclusion: aggression reflects interactions between psychological self-regulation, interpersonal relationships, organizational environments, and broader social influences rather than the effect of isolated risk factors.

A first theme emerging from the Research Topic concerns the importance of self-regulatory processes. Wang L. et al. showed that sport motivation was associated with lower aggression indirectly through emotional intelligence and self-control, highlighting the protective role of psychological resources. Likewise, Yilmaz et al. demonstrated that the relationship between physical activity and aggression was mediated by state anger, indicating that behavioral outcomes depend on how emotional experiences are regulated rather than on participation in physical activity itself. Together, these findings are consistent with Emotion Regulation Theory, which emphasizes that adaptive functioning depends on individuals' capacity to monitor and regulate emotional responses (Gross, 2015).

A complementary perspective is offered by Tan et al., whose findings reinforce the importance of resilience as a protective resource under demanding conditions (reduced resilience was associated with aggressive behaviors). Rather than eliminating stress, resilience enables individuals to adapt successfully to adversity and maintain effective functioning, supporting previous conceptualizations of resilience in sport (Sarkar and Fletcher, 2014). At the same time, Niţă et al., investigating sports coaches, extend current understanding by demonstrating that implicit aggressive tendencies may operate beyond conscious awareness, suggesting that both automatic and reflective processes contribute to behavioral regulation.

The evidence also challenges a long-standing assumption in sport sciences—that sport participation is inherently protective against aggression. The systematic review and meta-analysis conducted by Wang H. et al. reported that sport interventions significantly reduced hostility among adolescents, whereas their effects on aggression and anger were inconsistent and depended on intervention characteristics, sport type, and contextual factors. These findings suggest that sport itself is not inherently protective. Rather, its developmental value depends on the quality of the motivational, emotional, and social experiences it provides. This interpretation is closely aligned with Self-Determination Theory, which argues that supportive environments satisfying individuals' needs for autonomy, competence, and relatedness promote healthier motivation, self-regulation, and prosocial behavior (Ryan and Deci, 2017).

Taken together, these findings shift the focus from asking whether sport or education reduces aggression to understanding how these environments shape behavioral development. Psychological regulation represents only one component of a broader system in which relationships, institutional practices, and social contexts continuously influence individual behavior. Understanding these interactions is therefore essential for explaining aggression and for designing effective prevention strategies across both educational and sporting settings.

This ecological perspective is particularly evident in educational settings. Analyzing data from more than 16,000 schools across 75 countries, Ilgan et al. demonstrated that participation in extracurricular activities does not automatically reduce aggressive behavior or school misconduct. Rather, positive outcomes depend on school climate, instructional quality, and institutional effectiveness. These findings suggest that educational interventions should move beyond increasing participation alone and instead prioritize supportive learning environments in which positive relationships, fairness, and psychological safety become integral components of everyday educational practice.

A similar conclusion emerges from organized sport. Pereira-Vargas et al. showed that aggression directed toward referees reflects more than isolated emotional reactions during competition, and violence against officials is a multifaceted organizational issue involving athletes, coaches, spectators, clubs, and governing bodies. When abusive behaviors are inconsistently addressed or tacitly tolerated, they become embedded within the culture of competition, undermining both ethical values and the educational role of sport.

The organizational dimension of aggression is further highlighted by Greither et al., whose investigation of interpersonal violence in German sports clubs revealed a high prevalence of psychological, physical, non-contact and contact forms of sexual violence. Importantly, experiences of violence inside sport frequently co-occurred, while experiences of interpersonal violence inside and outside sport are related, “implying an increased risk of revictimization in either context.” Therefore, aggression develops across interconnected social systems rather than within isolated environments. These findings reinforce growing international recognition that safeguarding should be regarded as a fundamental component of sport governance rather than solely a response to individual incidents. This interpretation is further supported by the recent International Olympic Committee (IOC) consensus statement on interpersonal violence and safeguarding in sport (Tuakli-Wosornu et al., 2024), which conceptualizes safeguarding as a shared organizational responsibility rather than a reactive response to isolated incidents. The statement highlights the importance of integrated governance, education, athlete-centered policies, transparent reporting systems, and accountable leadership in creating safe sporting environments. From this perspective, preventing aggression extends beyond modifying individual behavior to fostering organizational cultures that consistently uphold respect, inclusion, dignity, and psychological safety.

The expansion of digital communication further illustrates the ecological complexity of aggression. Ruan et al. showed that cyber reactive aggression is influenced by hostile attribution bias, revenge motivation, and trait anger, with different pathways emerging for men and women. These findings indicate that online environments constitute a distinct social ecology in which anonymity, reduced interpersonal accountability, and rapid information exchange may amplify hostile cognitions while weakening self-regulatory processes. Consequently, cyber aggression should not be considered a secondary extension of offline behavior but an integral dimension of contemporary educational and sporting experiences.

Building on these converging findings, an Ecology of Aggression Framework is proposed, which conceptualizes aggression as the product of interactions across five interconnected ecological levels: individual, interpersonal, organizational, digital, and societal. At the individual level, behavioral regulation depends on emotional intelligence, motivation, resilience, self-control, anger regulation, and implicit cognitive processes. These capacities develop within interpersonal relationships involving teachers, coaches, peers, parents, officials, and teammates, while organizations establish the structural conditions that either encourage or discourage respectful interactions through leadership, safeguarding policies, and institutional climate. Increasingly, these processes are shaped by digital environments, whose influence is embedded within broader cultural values, public policies, media narratives, and social expectations surrounding competition, authority, and violence.

Collectively, the nine studies included in this Research Topic demonstrate that seemingly different forms of aggression share common psychological, relational, and organizational foundations. Recognizing these shared mechanisms shifts scientific attention from identifying isolated risk factors toward understanding the ecological conditions that foster healthy development. Future research should therefore adopt longitudinal and interdisciplinary approaches capable of examining reciprocal influences across ecological levels while informing prevention strategies that integrate psychological education, positive organizational cultures, safeguarding policies, and responsible digital engagement.
